# The influence of negative emotions on adolescents' pathological gaming: the role of self-control in the associations with aggression, academic stress, and pathological gaming

**DOI:** 10.3389/fpsyg.2025.1539162

**Published:** 2025-07-02

**Authors:** Jeong Ae Kim, Jae In Choi, Sung Je Lee, Eui Jun Jeong

**Affiliations:** ^1^Department of Humanities Counseling & Therapy, Konkuk University, Seoul, Republic of Korea; ^2^Department of Digital Culture & Contents, Konkuk University, Seoul, Republic of Korea

**Keywords:** pathological gaming, social intelligence, self-control, aggression, negative emotions, adolescent gamers, longitudinal study

## Abstract

**Introduction:**

Long-term exposure to negative emotions (e. g., anxiety, depression, loneliness) in adolescents has been reported to inhibit psychological growth and contribute to academic stress, aggression, and problem behaviors, including pathological gaming. However, there is a lack of longitudinal studies that examine the pathways through which academic stress and aggression, induced by negative emotions, lead to pathological gaming. This study aims to explore whether negative emotions significantly influence academic stress, self-control, and aggression, and whether self-control can mediate these pathways.

**Method:**

This study analyzed 3 years of longitudinal data from Korean adolescent gamers (*N* = 968) using structural equation modeling. It aimed to explain how self-control mediates the pathways between negative emotions, academic stress, aggression, and pathological gaming, and to investigate the associations among these factors.

**Results:**

Negative emotions were found to have a significant positive association with academic stress and aggression, but did not significantly associate with self-control. Furthermore, self-control was found to fully mediate the relationship between academic stress and pathological gaming, and partially mediate the relationship between aggression and pathological gaming. Therefore, while negative emotions did not directly influence self-control, they may affect other variables that, in turn, influence self-control, ultimately leading to pathological gaming. This finding is a key result of our study.

**Discussion:**

Preventing long-term exposure to negative emotions in adolescents and managing factors that influence self-control could be useful strategies for preventing pathological gaming. The results of this study suggest that continuous care for adolescents' psychological wellbeing and providing support to strengthen self-control could be effective interventions to suppress problem behaviors and foster healthy development in adolescents.

## 1 Introduction

Pathological gaming refers to excessive and persistent involvement in gaming, despite emerging social and psychological problems, ultimately leading to impaired self-regulation (Ferguson and Ceranoglu, 2014; Lemmens et al., 2011; Smith et al., 2015). Despite ongoing debates about the terminology, many researchers agree that excessive or compulsive gaming can lead to problematic behaviors (Lemmens et al., 2009), and empirical research continues to accumulate evidence of the negative consequences of pathological gaming (Nakayama et al., 2017; Zhuang et al., 2023). Specifically, pathological gaming has been associated with negative psychological issues such as anxiety, depression, social phobia, and fear (González-Bueso et al., 2018). Following this, in 2018, the World Health Organization (WHO) officially included “gaming disorder” in the 11th Revision of the International Classification of Diseases (ICD-11; World Health Organization, 2020), which has led to increased research aimed at understanding the mechanisms of pathological gaming and minimizing its negative effects (Pontes et al., 2021; Rafiemanesh et al., 2023).

Meanwhile, previous studies have identified various factors influencing pathological gaming, such as self-control, aggression, and academic stress. Research has also highlighted the mediating effects of self-control or aggression in the relationship between academic stress and pathological gaming. Recently, studies have explored the association between negative emotions and pathological gaming. However, longitudinal research exploring the interrelationships among negative emotions, self-control, aggression, and stress within an integrated framework remains limited. Thus, this study aims to identify the pathways through which negative emotions affect adolescents' pathological gaming through self-control, aggression, and stress, using longitudinal data.

## 2 Literature review and hypothesis development

According to prior research, adolescents are generally considered to exhibit higher impulsivity and weaker self-regulation compared to adults, which may make them more susceptible to problematic behaviors such as gambling and pathological gaming (Ha et al., 2007; Chambers and Potenza, 2003; Kim and Kim, 2015). In addition, adolescents with higher levels of pathological gaming have been reported to show lower social skills, greater impulsivity, and a higher likelihood of experiencing mental health concerns such as depression and anxiety, as well as lower academic achievement (Gentile et al., 2011). In particular, adolescents who experience difficulties in psychological development and social relationships may be less able to adapt to rapid changes, and such difficulties could persist over time (Lemmens et al., 2011; Wagner and Anthony, 2002; Wartberg et al., 2019). Therefore, examining the processes that may contribute to pathological gaming in adolescents and identifying potentially associated factors appears to be important for broadening the understanding of digital behavior issues among this population.

### 2.1 Negative emotions and pathological gaming

Pathological gaming is known to be triggered through the dynamics between various psychological factors such as anxiety, loneliness, depression, and aggression (Carli et al., 2012; González-Bueso et al., 2018; Lemmens et al., 2011). Multiple studies support the fact that individuals with pathological gaming tend to have more negative emotions or issues with emotional regulation compared to those without pathological gaming (Lin P. Y. et al., 2020; Ma and Sui, 2023). For example, anxiety, depression, and loneliness have been significantly associated with pathological gaming (Fazeli et al., 2020; Lemmens et al., 2011). Adolescents overwhelmed by negative emotions may be more exposed to stress and may even experience negative impacts on their psychological development (Herts et al., 2012; King et al., 2011; Ma and Sui, 2023). Additionally, individuals overwhelmed by negative emotions may immerse themselves deeply in gaming as a way to fulfill unmet psychological needs or to escape negative states such as loneliness, boredom, or anxiety (Li et al., 2021; Maroney et al., 2019).

Considering these findings, negative emotions not only influence the creation of negative states like stress, but can also stimulate aggression, weaken self-control, disrupt normal development, and even encourage problematic behaviors such as pathological gaming (Hong et al., 2020; Li et al., 2021; Kessler et al., 2001). For example, prolonged exposure to negative emotions can lead to the accumulation of stress and increase sensitivity to aggression (Herts et al., 2012; Repetti et al., 2002). Related studies have confirmed that adolescents with higher levels of pathological gaming also show higher levels of depression and anxiety, as well as more interpersonal problems and aggressive behaviors (Milani et al., 2018; Torres-Rodríguez et al., 2018). Therefore, it is essential to examine, through longitudinal data, how negative emotions influence elements such as stress, aggression, and self-control, which contribute to adolescents' pathological gaming.

#### 2.1.1 Anxiety

Anxiety refers to persistent and excessive worry that leads to physiological tension and negative mood, interfering with normal daily activities (Gale and Oakley-Browne, 2004; Teychenne et al., 2015). High levels of anxiety are associated with cognitive issues such as fatigue, reduced memory and concentration, fear, and an increased risk of suicidal attempts, as well as physical impairments and addictive behaviors (Zender and Olshansky, 2009). In particular, anxiety during childhood and adolescence is known to cause problems such as depression, emotional regulation issues, impaired social skills, and disruption in peer relationships (Axelson and Birmaher, 2001; Nieuwenhuijsen et al., 2003; Rapee et al., 2009). It not only induces high levels of stress but also has a negative impact on the promotion of aggressive behaviors (Herts et al., 2012; Lansford et al., 2010). For example, a study of 973 adolescents found that those with higher social anxiety scores were more likely to have lower social skills and face peer issues (Inderbitzen et al., 1997). Other studies have shown that adolescents with higher anxiety scores are more likely to have negative self-images and experience suicidal thoughts, peer victimization, isolation, and loneliness (Biswas et al., 2020; Di Blasi et al., 2015). Additionally, other research has found that adolescents with social anxiety have significant associations with reactive and relational aggression (Andrews et al., 2019). This study specifically noted that adolescents exposed to high levels of anxiety are more likely to have emotional regulation issues and are likely to respond to high stress and intense negative emotions in inefficient and aggressive ways.

Anxiety is also closely associated with pathological gaming. For example, individuals with high levels of social anxiety tend to immerse themselves more easily in the anonymous online gaming environment, where social relationships can be restructured more easily and communication is less challenging compared to face-to-face situations (Gioia et al., 2022; Hussain and Griffiths, 2009; Wei et al., 2012). However, the social rewards provided by online gaming are limited, and the anxiety-reducing effects are often temporary, potentially increasing social anxiety in the long term as real-life relationships are neglected (Gioia et al., 2022). Prior research has shown that anxiety increases pathological gaming (Ferguson et al., 2022), and multiple studies have also reported a significant correlation between pathological gaming and anxiety (Karaca et al., 2020; Milani et al., 2018).

#### 2.1.2 Loneliness

Unlike social isolation, which refers to the objective lack of social interactions, loneliness is a subjective feeling of being alone (Hwang et al., 2020). More specifically, loneliness is the degree of perceived deficiency in the quantity or quality of relationships with others or society, and it is an unpleasant and distressing psychological experience that results from interpersonal conflicts or imbalances (Karaoglan Yilmaz et al., 2022). Loneliness often co-occurs with other negative emotions such as anxiety and depression, and it can negatively impact mental health or serve as a precursor to problematic behaviors such as externalizing behaviors (Check et al., 1985; Okruszek et al., 2020). In a longitudinal study conducted with British university students, it was found that higher loneliness scores were associated with increased anxiety, depression, and stress (Richardson et al., 2017). Similarly, an online survey conducted during the COVID-19 period with 1,008 participants reported a significant correlation between loneliness and mental health issues such as anxiety and depression (Horigian et al., 2021).

Adolescents, due to their physical, cognitive growth and self-concept confusion, and the rapid changes in social relationships, are particularly vulnerable to loneliness. They are more likely to be exposed to social failures or conflict experiences, making them more susceptible to loneliness than adults (Stickley et al., 2014). Importantly, loneliness can influence the development of negative traits in adolescents, trigger stress, and lead to problematic behaviors (Marsh et al., 2011; Stickley et al., 2014). For example, adolescents experiencing loneliness may engage in more aggressive and violent behaviors to protect themselves in social conflicts (Marsh et al., 2011). In a longitudinal study conducted with adolescents, it was found that those who experienced chronic loneliness were more likely to exhibit lower social skills and higher aggression scores later on Schinka et al. (2013). Additionally, a study of 1,510 Spanish adolescents confirmed that loneliness, along with depression, was significantly associated with aggressive behaviors (Estévez López et al., 2018). Furthermore, another study found that adolescents with chronic loneliness had higher depression and anxiety scores, as well as higher perceived stress compared to their peers (Vanhalst et al., 2013). Another online survey involving 843 college students also found that loneliness was a significant factor influencing both aggressive behaviors and smartphone addiction (Karaoglan Yilmaz et al., 2022).

Loneliness is known to be one of the factors that contribute to pathological gaming (Çelik and Odaci, 2013). The unique features of online gaming can be an attractive means for individuals experiencing loneliness to fulfill their unmet needs. For instance, the anonymous online environment may offer a chance for individuals facing social difficulties or introverted individuals to escape the adverse conditions and fears of real-world social relationships and form new social connections (Amichai-Hamburger and Ben-Artzi, 2003; Bremer and Rauch, 1998; Odaci and Çelik, 2013). In other words, adolescents with poor social skills and experiencing loneliness may become overly immersed in virtual social relationships and communities to temporarily alleviate their negative emotions (Lemmens et al., 2011). In this regard, one study found that loneliness is positively correlated with adolescents' pathological gaming and mediates the relationship between self-esteem and pathological gaming (Zeng et al., 2016).

#### 2.1.3 Depression

Depression refers to exaggerated and prolonged feelings of sadness, helplessness, negative cognition, and emotions such as frustration and fatigue, which can persist for weeks and interfere with daily life, constituting an affective disorder or negative emotion (Lin J. et al., 2020; Mamani-Benito et al., 2022). Depression has been shown to negatively effect on adolescent development and is associated with issues such as decreased educational attainment, damaged social relationships, substance abuse, mental health problems, and an increased risk of suicide (Copeland et al., 2014; Keles et al., 2020).

Previous research suggests that high levels of depression can make individuals more vulnerable to stress (Kendler and Gardner, 2016). For instance, individuals with high levels of depression may be less motivated to improve their mood and may prefer to remain in a sad or pessimistic state, which makes them less active in coping with stress and more vulnerable to its negative effects (Mamani-Benito et al., 2022). Additionally, contrary to the common belief that feelings of helplessness and sadness would serve as opposite indicators of aggression, empirical studies suggest that depression may actually trigger general aggression, relational aggression, and self-directed aggression (Dutton and Karakanta, 2013; Roland, 2002). Previous research has found that depression can distort attributions of negative emotions or events, leading individuals to blame others for causing problems, which may be further amplified when combined with other negative emotions like loneliness (Dutton and Karakanta, 2013).

Depression is closely associated with pathological gaming. Depression enhances negative self-beliefs and emotions, which may influence individuals to avoid self-care or external activities, opting instead to over pathological gaming as a means of escape (Scerri et al., 2019). In a study conducted with 1,401 adults in Korea, it was found that pathological gaming was associated with depression and mood regulation issues (Kim et al., 2017). Specifically, the study indicated that gaming disorder could be promoted by motivations to escape negative emotions such as anxiety, sadness, and anger. Additionally, a survey of 161 middle school students found that depression was a significant factor in explaining pathological gaming (Li et al., 2021). The study reported that adolescents overwhelmed by depression may immerse themselves excessively in gaming as a way to escape their real-world self-image and seek an idealized self, leading to pathological gaming.

### 2.2 Stress, aggression, and pathological gaming

Adolescents' negative emotions not only lead to stress, distorted judgment, or behaviors but can also pose long-term risks to their psychological development (Herts et al., 2012; Repetti et al., 2002). Furthermore, intensified negative psychological traits such as stress and aggression may hinder the development of psychological factors like self-control, which suppress pathological gaming and can serve as direct contributors to problematic behaviors (King et al., 2011; Milani et al., 2018; García-Sancho et al., 2014).

#### 2.2.1 Stress and pathological gaming

Stress is a negative emotional and physiological response that occurs when individuals perceive that the demands or challenges they face exceed their ability to cope or control (Cohen et al., 2007). High levels of stress can overwhelm individuals with negative feelings and physiological arousal, which can lead to cognitive and behavioral responses that are maladaptive, potentially impacting mental health in the long term (Rezaei and Mousanezhad Jeddi, 2020). For example, stress can activate the nervous system, induce negative moods, and lead individuals to interpret neutral stimuli negatively, making them more impulsive or aggressive (Estévez López et al., 2018; Liu and Boyatzis, 2021).

Adolescence is a period marked by heightened sensitivity in psychological and social relationships, so the consequences of stress are likely to be more threatening during this stage (Blakemore and Mills, 2014; Sisk and Gee, 2022). Negative emotions, such as those arising from frustration due to social disconnection or negative situations, are known to be key factors contributing to repeated stress exposure in adolescents (Herts et al., 2012; Kendler and Gardner, 2016; Repetti et al., 2002). For instance, negative situations, such as social exclusion or frustration, can serve as long-term stressors. Adolescents exposed to prolonged stress are more likely to be vulnerable to negative mental health outcomes and problematic behaviors compared to their peers (Panier et al., 2022; Sontag et al., 2011).

Adolescents' stress can be broadly divided into social stress and academic stress. Due to the time adolescents spend in school, they are particularly exposed to social and peer-related stress. In addition, academic stress, which results from the pressure to perform academically, is one of the most common forms of stress experienced during this period (Aloia and McTigue, 2019). Psychological pressure related to social relationships and achievement within the school environment can negatively affect adolescents' mental health and development if it persists unnecessarily for extended periods (Alzahem et al., 2013; Knowles et al., 2008; Špiljak et al., 2022). In a study involving Korean adolescents, high levels of academic stress were found to negatively affect self-control and also influenced pathological gaming (Jeong et al., 2019).

Excessive stress is known to be one of the factors contributing to pathological gaming. A study found that there was a significant positive correlation between academic stress and addiction to shooting games among adolescents (Mun, 2023). Previous studies have shown that high levels of stress may prompt adolescents to adopt avoidance-based coping strategies, which could lead to an increased tendency to over-immerse themselves in gaming, a maladaptive alternative to active problem-solving, contributing to pathological gaming (King et al., 2011; Milani et al., 2018). For example, adolescents exhibiting high levels of stress may engage in pathological gaming as a maladaptive coping strategy to regulate negative emotions or to compensate for perceived deficiencies in their offline environment (Caplan et al., 2009; Milani et al., 2018; Snodgrass et al., 2013).

#### 2.2.2 Aggression

Aggression refers to all behaviors performed with the intent to harm others, including verbal and physical aggression, cognitive hostility, and emotional elements such as anger (Buss and Perry, 1992; Chen et al., 2014; García-Sancho et al., 2014). Previous research has indicated that aggression is often exacerbated by negative psychological states such as anxiety and loneliness. For instance, interpersonal conflicts or threats to social reputation can elevate individuals' anxiety and damage emotional regulation, which may trigger aggressive behaviors (Loudin et al., 2003; Kashani et al., 1991; Neumann et al., 2010). Loneliness and depression are also known to serve as negative internalized events that can trigger aggressive behaviors (Blossom and Apsche, 2013). Individuals with high levels of loneliness are more likely to perceive others' intentions and behaviors negatively, avoid confronting social conflicts constructively, and escalate interpersonal disputes (Anderson et al., 1983; Yavuzer et al., 2019).

Aggression has been identified as a risk factor for impulsivity, emotional regulation difficulties, and problematic behaviors such as addiction and suicide (Chen et al., 2014; Lansford, 2018; McCloskey and Ammerman, 2018; Naaijen et al., 2020; Piko and Pinczés, 2014; Rothenberg et al., 2019). In particular, it has been closely linked to pathological gaming. For example, individuals with high aggression levels are more likely to become excessively involved in violent games, as a way of satisfying aggressive urges (Griffiths, 2000; Kim et al., 2008). Additionally, several studies have reported that aggression and loneliness are key precursors to pathological gaming (Odaci and Çelik, 2013).

### 2.3 Self-control and pathological gaming

Self-control refers to the ability to resist impulses or temptations to perform certain behaviors (Kim et al., 2008). In this context, self-control can be understood as the ability to guide and regulate one's actions. Conversely, a lack of self-control is closely related to the tendency to engage in activities that provide immediate gratification without considering long-term goals (Sumiyana et al., 2022). Self-control is a key factor necessary for healthy adolescent development and happiness, as it not only helps suppress negative moods or emotions but also influences behavior and judgment toward avoiding losses or seeking positive rewards, thereby increasing life satisfaction and happiness (Cheung et al., 2014). On the other hand, a lack of self-control is strongly associated with negative emotions such as anxiety, stress, and aggression, and it is reported to increase vulnerability to problematic behaviors (Sumiyana et al., 2022). In addition, parenting styles may influence adolescents' use of video games (Cote et al., 2021; Bonnaire and Phan, 2017). Recent research suggests that lower levels of parental monitoring are associated with poorer interpersonal skills, particularly higher impulsivity and stress in social situations, and adolescents with higher levels of impulsivity may be more likely to use video games as a means of escaping from reality (Commodari et al., 2024).

Generally, self-control is considered a protective factor that helps mitigate the harmful effects of negative events or emotions. However, high levels of stress and negative traits can hinder the development of self-control. Some studies suggest that self-control, much like a muscle, is not unlimited but rather limited, and gradually depletes as individuals regulate certain habits, behaviors, rituals, and reactions (Baumeister et al., 1998; Oaten and Cheng, 2005). For example, individuals exposed to situations that trigger cognitive distortions, such as anger, depression, or stress, may initially exert self-control to resist engaging in problematic behaviors. However, when their resources are depleted, they may fail to regulate their behavior. This suggests that long-term and repeated exposure to negative emotions, stress, and negative psychological traits can have a detrimental effect on the development or proper functioning of self-control.

For example, research on rumination—repetitive thinking about negative events—has suggested that maladaptive anger regulation and negative emotional states may deplete the cognitive resources necessary for self-control, thereby increasing the likelihood of aggressive behavior (Denson et al., 2012, 2011). Similarly, other studies have reported that chronic stress can impair self-regulatory capacity, particularly during periods of heightened academic stress such as exam seasons, when emotional regulation and self-control are more vulnerable in adolescents (Oaten and Cheng, 2005). Negative emotions such as loneliness and depression have also been identified as significant predictors of diminished self-control (Özdemir et al., 2014). According to these studies, individuals experiencing negative mood states may feel a strong urge to regulate their emotions, which can lead them to engage in behaviors aimed at obtaining immediate rewards or escaping from distress—patterns that may contribute to the development of problematic behaviors (Caplan, 2003; Özdemir et al., 2014; LaRose et al., 2003). In other words, negative emotions such as loneliness and depression may interfere with adaptive emotion regulation, thereby disrupting the normal functioning of self-control.

Previous research has indicated that adolescents' pathological gaming is closely associated with a lack of self-control. High levels of self-control help prevent adolescents from being swept up in short-term pleasures and rewards, thus preventing pathological gaming. Conversely, a lack of self-control allows for impulsive behavior, which is known to contribute to addictive behaviors (Kim et al., 2008). Several studies have reported that individuals with high levels of pathological gaming tend to have lower self-control and emotional regulation skills compared to others, and research has shown that self-control is a strong protective factor against pathological gaming behaviors (Kim et al., 2008; Jeong et al., 2019; Ji et al., 2022). A study involving 3,034 children from multiple countries also confirmed that higher gaming addiction scores were associated with higher impulsivity (Gentile et al., 2011).

**Figure 1:**
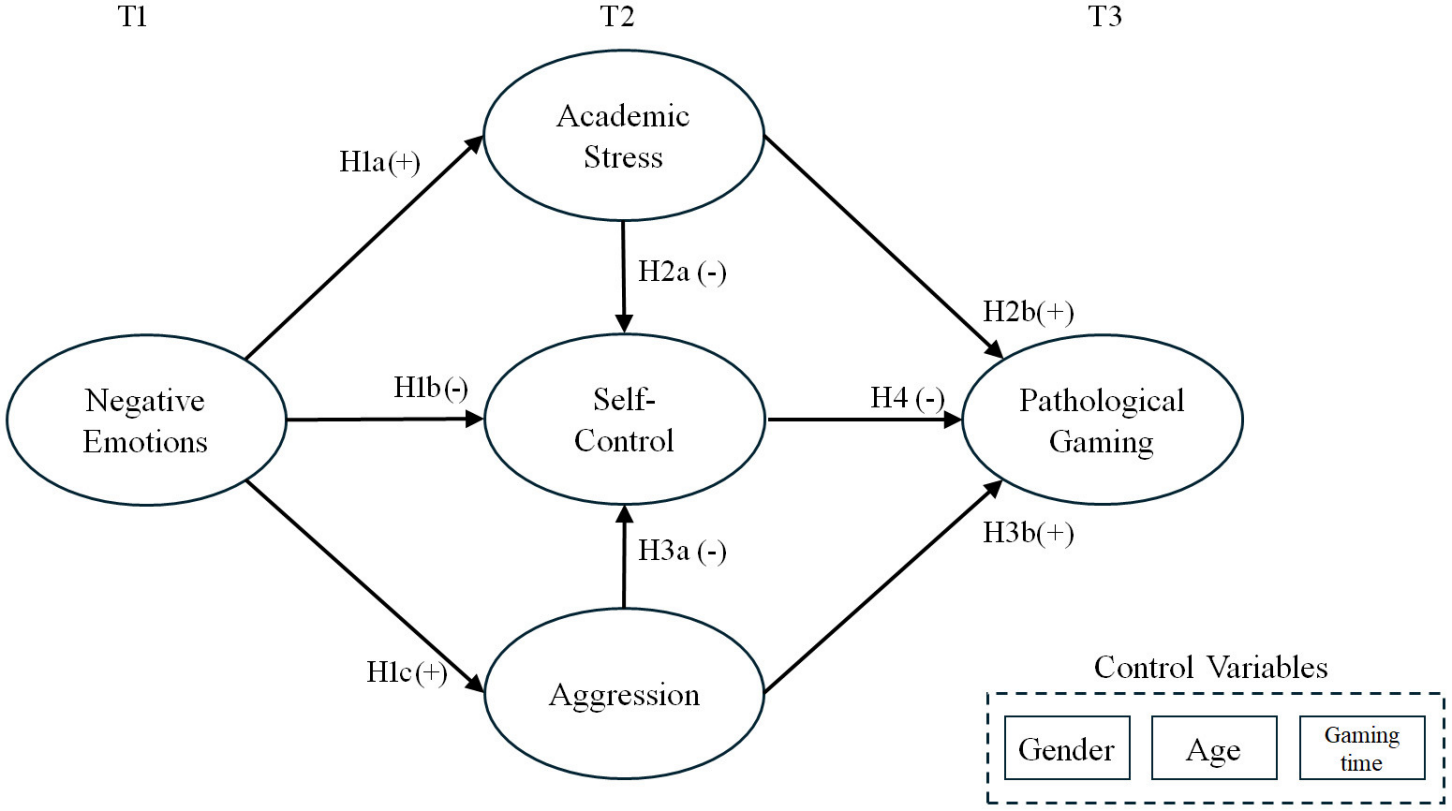
Research model.

Thus, this study aims to empirically validate a path model, using longitudinal data, to explore how adolescents' negative psychological factors affect self-control and, in turn, how this influences their pathological gaming (refer to Figure 1). To test this model, the following hypotheses were developed:

H1a. Adolescents' negative emotions will be positively associated with academic stress.
H1b. Adolescents' negative emotions will be negatively associated with self-control.
H1c. Adolescents' negative emotions will be positively associated with aggression.
H2a. Adolescents' academic stress will be negatively associated with self-control.
H2b. Adolescents' academic stress will be positively associated with pathological gaming.
H3a. Adolescents' aggression will be negatively associated with self-control.
H3b. Adolescents' aggression will be positively associated with pathological gaming.
H4. Adolescents' self-control will be negatively associated with pathological gaming.
H5. Adolescents' self-control will mediate the relationship between academic stress and pathological gaming.
H6. Adolescents' self-control will mediate the relationship between aggression and pathological gaming.

## 3 Methods

### 3.1 Data collection

In this study, we utilized panel data from the Korean Adolescent Game User Cohort Research, conducted by the Korea Creative Content Agency (KOCCA) between 2015 and 2018 to assess the gaming behavior of primary, middle, and high school students. The panel data collection was approved by the Institutional Review Board (IRB) of the partner institution, Konkuk University (IRB No. 7001355-201408-HR-031). Informed consent was obtained from the participants, ensuring privacy and anonymity regarding personal data related to the survey. A quota sampling method was used based on grade and gender proportions. Trained professional interviewers conducted face-to-face interviews across three times (T1, T2 and T3) of a cohort study, following established survey protocols to collect responses, the intervals between T1, T2, and T3 were each 1 year. The same questionnaire was administered throughout all waves. Each panel participant was compensated with USD 27.00 for their participation. Detailed descriptions of the survey methodology and data are available on the KOCCA website (www.kocca.kr, accessed October 1st, 2023).

For data collection, this study measured adolescents' levels of negative emotions (e.g., anxiety, depression, and loneliness) at T1. At T2, academic stress, self-control, and aggression were assessed, followed by the measurement of pathological gaming at T3. A total of 1,521 adolescents participated at T1, but this number decreased to 1,158 at T2 and 968 at T3, resulting in a total attrition of 584 participants over the study period. Final analyses were conducted using a complete case analysis approach, including only those who provided valid responses to all relevant variables at each wave (T1, T2, and T3). This approach was adopted to ensure the temporal consistency of the hypothesized causal pathways and to enhance the internal validity of the structural model.^1^

Thus, for the analysis, data from 968 students who participated in the survey were used. Of the total participants, 477 were male (49.3%) and 491 were female (50.7%). In terms of school levels, there were 345 elementary school students (35.6%), 333 middle school students (34.3%), and 290 high school students (30%). Students were questioned and responded regarding their gaming habits and their usual thoughts about gaming. Table 1 summarizes the demographic characteristics of the respondents.

**Table 1:** Demographic characteristics.

**Characteristics**		**All participants (968)**	
		**Frequency**	**(%)**
Gender	Male	477	49.3
	Female	491	50.7
Age group	Elementary group	345	35.6
	Middle school	333	34.4
	High school	290	30
Online game duration (daily average)	Not playing	100	10.3
	Under 30m	198	20.5
	30m−1H	213	22
	1H−2H	205	21.2
	2H−3H	134	13.8
	3H−4H	68	7
	4H−5H	22	2.3
	5H−6H	10	1
	Over 6H	18	1.9

### 3.2 Measurement

To address the research questions, this study employed partial least squares structural equation modeling (PLS-SEM) to explore the causal relationships among variables, focusing on the explanatory power of the proposed model based on hypotheses derived from prior research. In addition, repeated measures analysis using the General Linear Model (GLM) was conducted. The survey included items assessing constructs such as negative emotions (e.g., anxiety, depression, and loneliness), academic stress, self-control, and aggression. The questionnaire items were largely drawn from validated instruments frequently cited in prior studies. Diverse Likert scales were applied to capture responses for each construct, except for gaming time, which was measured using a non-Likert scale approach.

**Anxiety** To measure anxiety, the Generalized Anxiety Disorder 7 item scale (GAD-7) developed by Spitzer et al. (2006) was used. The scale consists of six items rated on a Four-point Likert scale, ranging from “0 = not at all” to “3 = nearly every day.” Example items include: “I found it hard to control my worries,” “I was anxious about what might happen,” and “I worried too much about different things” (α = 0.890).

**Loneliness** refers to a “dissatisfying and unstable psychological state resulting from a lack of social interaction” (Perlman and Peplau, 1982). It is generally associated with social isolation, but individuals can feel lonely even when surrounded by others. Therefore, loneliness is more related to the qualitative rather than the quantitative aspects of social relationships (Perlman and Peplau, 1982). The UCLA Loneliness Scale was used to measure the degree of loneliness. The scale consists of 10 items rated on a Four-point scale from “1 = not at all” to “4 = very often.” Items include: “There is no one I can count on in times of need,” “I feel lonely,” and “I feel that I can rely on others” (α = 0.912).

**Depression** is characterized by symptoms such as sadness, loss of interest or pleasure, feelings of guilt or low self-esteem, sleep disturbances, loss of appetite, fatigue, and concentration problems (World Health Organization). To measure depression, the CESD-11 (Center for Epidemiological Studies Depression Scale-11), a shortened version of the original 20-item CESD developed by the National Institute of Mental Health, was used. The scale consists of 11 items rated on a Four-point scale from “0 = rarely or none of the time” to “3 = most of the time or all of the time.” Items include: “I felt sad,” “I had trouble sleeping,” and “I felt that others disliked me” (α = 0.883).

**Academic stress** was measured using a 4 item subscale from the Life Stress Scale developed by Kim and Chon (1993). Items include: “I could not improve my grades despite putting in effort,” “I had trouble focusing on studying,” and “I felt isolated by my peers.” The scale was rated on a Three-point scale from “0 = not at all” to “2 = often” (α = 0.794).

**Self-control** is defined as the ability to choose a delayed, higher-value reward over an immediate, less valuable option (Kirk and Logue, 1996). It is sometimes used interchangeably with terms such as delay of gratification, effortful control, willpower, executive control, time preference, self-discipline, self-regulation, and ego strength (Duckworth, 2011). To measure self-control, the Brief Self-Control Scale (BSCS) developed by Morris et al. (2004) was used, for which validation studies have also been conducted in South Korea (Hong et al., 2012). The scale consists of 13 items rated on a Five-point Likert scale from “1 = not at all true” to “5 = very true.” Example items include: “I don't often give in to temptation,” “Sometimes I fail to finish tasks because I get too distracted by fun things,” and “I often act without thinking about other possible solutions” (α = 0.810).

**Aggression** refers to “behavior performed with the intent to harm others” (Anderson and Bushman, 2002). This includes not only physical and verbal aggression but also cognitive hostility and emotional anger (Berkowitz, 1993). To measure aggression, the Short-Form Buss-Perry Aggression Questionnaire (BPAQ-SF) was used. The BPAQ-SF was developed by Diamond et al. (2005) by modifying the original 29-item Buss-Perry Aggression Questionnaire (BPAQ) by Buss and Perry (1992). The scale consists of 12 items rated on a Five-point Likert scale from “1 = not at all true” to “5 = very true.” Subscales include physical aggression, verbal aggression, anger, and hostility. Example items include: “I frequently clash with others,” “I sometimes get angry for no reason,” and “I struggle to control my anger” (α = 0.880).

**Pathological gaming** Although there is no universally agreed-upon definition of pathological gaming, it is generally understood to refer to the obsessive or excessive use of gaming that results in negative consequences such as physical and mental health problems or negative outcomes in academic, professional, or social functions. Pathological gaming is often associated with increased gaming time, sleep deprivation, and social relationship deficits (Allison et al., 2006). In the absence of a standardized diagnostic system, the Internet Addiction Test (IAT), originally developed by Dr. Kimberly Young for internet addiction, was adapted for gaming (Jin and Lee, 2022; Kim, 2021). This study used a modified version of the IAT that was adjusted for gaming contexts. The scale consists of 20 items rated on a Five-point Likert scale from “1 = not at all” to “5 = very true.” The items cover aspects such as salience, excessive use, neglecting responsibilities, neglecting social life, lack of self-control, and anticipation. Example items include: “I neglect other activities because of gaming,” “Gaming causes problems in my school life,” and “I feel bored and empty without gaming” (α = 0.94).

## 4 Results

### 4.1 Reliability and validity test

The data were analyzed using the PLS-SEM method. In the PLS-SEM statistical analysis, the measurement model was evaluated based on statistical criteria for convergent validity (e.g., factor loading values, AVE), internal consistency reliability (e.g., Cronbach's alpha value, CR), and discriminant validity. The acceptable thresholds for these measures were as follows: the extracted average variance (AVE) should be above 0.5; for higher internal consistency, Cronbach's alpha and the composite reliability (CR) for each construct should be at least 0.7; and discriminant validity was assessed using the Heterotrait-Monotrait ratio (HTMT), a measure that complements the Fornell-Larcker criterion, which has been criticized for lacking discriminant validity in general research environments. The HTMT ratio threshold for acceptable values is 0.9. Tables 2, 3 confirm that convergent validity, internal consistency reliability, and discriminant validity were achieved through the measurement model analysis. The data were analyzed using SmartPLS 3 software (v. 3.3.2, Bönningstedt, Germany).

**Table 2:** Results for measurement model.

**Scale/items**	**Cronbach's α**	***M***	***SD***	**CR**	**AVE**	**R2**
Negative emotions (NGA)/second order						
Anxiety (AXT)	0.89	0.46	0.543	0.89	0.645	0.638
Loneliness (LON)	0.912	1.56	0.553	0.917	0.619	0.669
Depression (DPR)	0.883	0.26	0.458	0.884	0.631	0.698
Academic stressors (ACS)/first order	0.794	0.67	0.566	0.81	0.706	0.082
Self-control (SCT)/first order	0.81	3.32	0.792	0.811	0.636	0.172
Aggression (AGR)/first order	0.88	1.8	0.773	0.886	0.627	0.172
Pathological gaming (PTG)/first order	0.94	2.24	0.978	0.942	0.705	0.264

M, Mean; SD, Standard deviation; CR, Composite reliability; AVE, Average variance extracted; R2, R Square adjusted.

**Table 3:** Heterotrait-Monotrait ratio (HTMT) for discriminant validity.

**Variables**	**PTG**	**AGR**	**NGA**	**ACS**	**SCT**
Pathological gaming (PTG)					
Aggression (AGR)	0.366				
Negative emotions (NGA)	0.304	0.456			
Academic stress (ACS)	0.268	0.425	0.334		
Self-control (SCT)	0.409	0.604	0.341	0.585	

Shaded boxes are the standard reporting format of PLS-SEM HTMT analysis.

### 4.2 Research model test

Based on the evaluation of the measurement model, the structural model analysis was conducted to test the hypotheses (refer to Table 4). Our hypotheses were statistically supported in the structural model, except for H1b and H2b. A summary of the hypothesis testing results is as follows:

**Table 4:** Results of the hypothesis tests.

**Hypothesis**	**Coef**.	**Mean**	***SD***	***t***	**Results**
H1a. Negative emotions (NGA) → Academic stressors (ACS)	0.288	0.289	0.03	9.678^***^	Accepted
H1b. Negative emotions (NGA) → Self-control (SCT)	−0.045	−0.046	0.031	1.459	Rejected
H1c. Negative emotions (NGA) → Aggression (AGR)	0.416	0.417	0.031	13.564^***^	Accepted
H2a. Academic stressors (ACS) → Self-control (SCT)	−0.339	−0.339	0.027	12.338^***^	Accepted
H2b. Academic stressors (ACS) → Pathological gaming (PTG)	0.064	0.064	0.034	1.895	Rejected
H3a. Aggression (AGR) → Self-control (SCT)	−0.373	−0.373	0.029	12.825^***^	Accepted
H3b. Aggression (AGR) → Pathological gaming (PTG)	0.165	0.165	0.035	4.759^***^	Accepted
H4. Self-control (SCT) → Pathological gaming (PTG)	−0.215	−0.215	0.036	5.963^***^	Accepted
H5. Academic stressors (ACS) → Self-control (SCT) → Pathological gaming (PTG)	0.073	0.073	0.014	5.215^***^	Accepted
H6. Aggression (AGR) → Self-control (SCT) → Pathological gaming (PTG)	0.08	0.08	0.014	5.569^***^	Accepted
[Control variable] Gender → Pathological gaming (PTG)	−0.499	−0.501	0.059	8.44^***^	–
[Control variable] Age → Pathological gaming (PTG)	−0.044	−0.044	0.031	1.423	–
[Control variable] Online game duration → Pathological gaming (PTG)	0.159	0.161	0.031	5.222^***^	–

Coef., Coefficient, Significant level: ^***^p < 0.001.

Adolescents' negative emotions (T1) were found to have a significant positive impact on academic stress (T2; β = 0.288, *p* < 0.001) but did not significantly affect self-control (T2; β = −0.045, *p* > 0.05). Adolescents' negative emotions (T1) were most strongly associated with aggression (T2; β = 0.416, *p* < 0.001).

Adolescents' academic stress (T2) significantly affected self-control (T2; β = −0.339, *p* < 0.001), but did not affect pathological gaming (T3; β = −0.064, *p* > 0.05). And, Adolescents' aggression (T2) had a significant negative impact on self-control (T2; β = −0.373, *p* < 0.001), and a significant positive effect on pathological gaming (T3; β = 0.165, *p* < 0.001).

Adolescents' self-control (T2) had a significant negative impact on pathological gaming (T3; β = −0.215, *p* < 0.001). Adolescents' self-control (T2) was found to mediate the relationship between academic stress (T2) and pathological gaming (T3; β = 0.073, *p* < 0.001), and between aggression (T2) and pathological gaming (T3; β = 0.080, *p* < 0.001).

In the model without self-control (T2), academic stress (T2) did not significantly affect pathological gaming (T3; β = 0.064, *p* < 0.001). However, in the model with self-control (T2), academic stress (T2) was found to significantly affect pathological gaming (T3; β = 0.073, *p* < 0.001), indicating a full mediation effect by self-control (T2).

**Figure 2:**
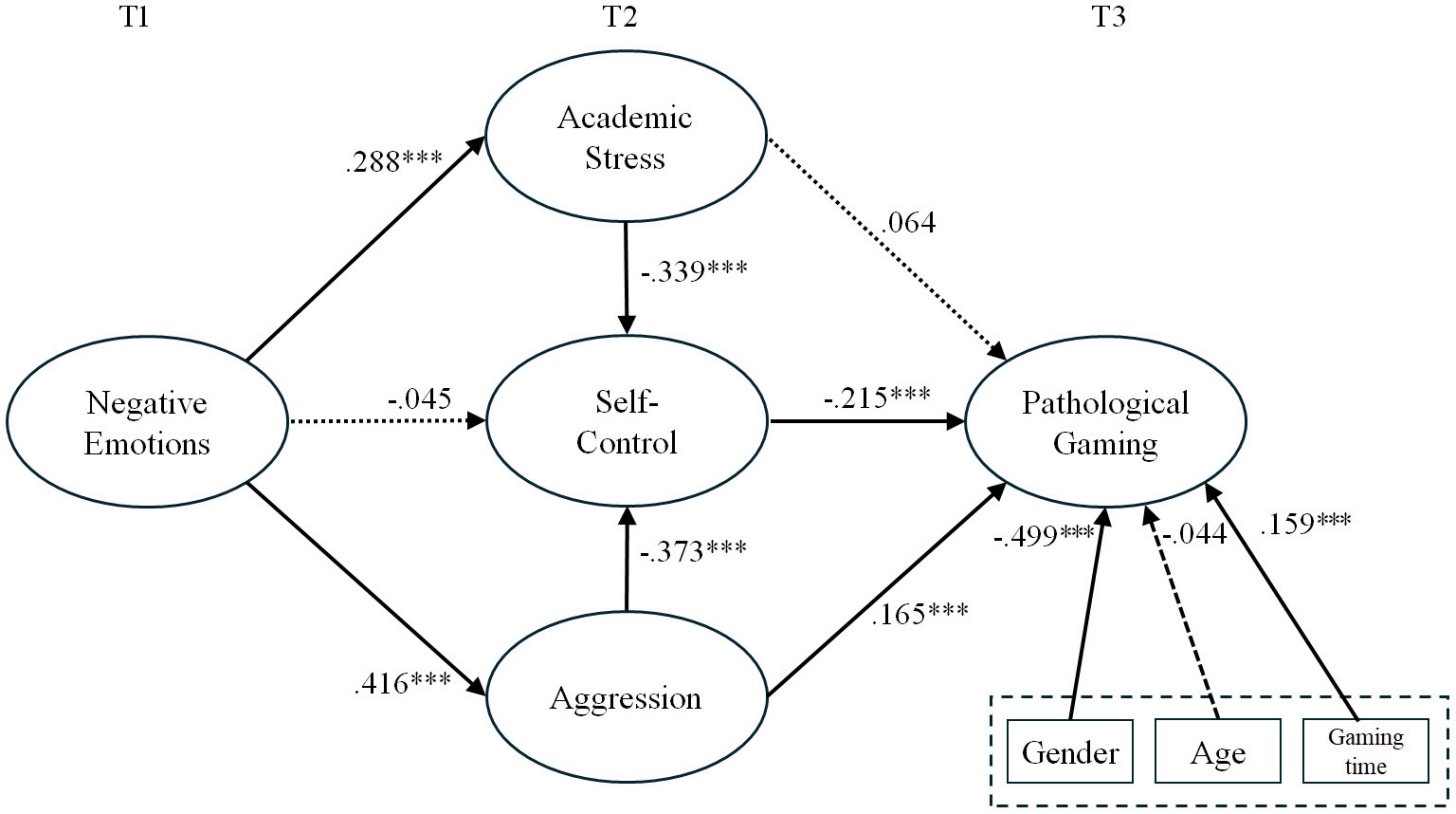
Research model and hypothesis. Significant level: ***p < 0.001.

In the model without self-control (T2) too, aggression (T2) significantly affected pathological gaming (T3; β = 0.165, *p* < 0.001). In the model with self-control (T2), aggression (T2) continued to significantly affect pathological gaming (T3; β = 0.080, *p* < 0.001), indicating a partial mediation effect (refer to Figure 2).

## 5 Discussion

### 5.1 Findings

This study examined how negative emotions, academic stress, self-control, and aggression influence the progression of adolescent gaming behavior into pathological gaming. We hypothesized that internal factors, such as negative emotions (e.g., anxiety, depression, and loneliness), may increase stress levels and aggression, while also undermining the development of self-control. These mechanisms, in turn, may contribute to the emergence of problematic behaviors, including pathological gaming (King et al., 2011; Milani et al., 2018; García-Sancho et al., 2014). The results of this study are generally consistent with prior research, and the implications are discussed as follows:

First, adolescents' negative emotions may be associated with aggression. Negative emotions such as anxiety or depression that emerge during adolescence may be developmental traits triggered by relationships with parents, peers, and teachers. However, prolonged exposure to negative emotions may lead to hostility or aggressive behaviors toward specific targets, which can act as a precursor to pathological gaming behaviors driven by stress.

Second, while our results did not show a significant direct effect of adolescents' negative emotions on self-control, the possibility that self-control may mediate the relationship between aggression and pathological gaming is noteworthy. This suggests that when negative emotions in adolescents lead to increased aggression, a decrease in self-control may lead to the development of pathological gaming.

Third, although academic stress did not significantly affect pathological gaming, self-control was found to have a significant effect on pathological gaming. This contrasts with prior discussions that have focused on external environmental factors, such as parental attitudes, peer groups, and teacher support, which were considered key contributors to pathological gaming. Simultaneously, negative emotions, such as anxiety, depression, and loneliness, as internal factors, may also contribute to pathological gaming through a pathway mediated by self-control.

As previously mentioned, based on a dual-system perspective, conditions such as ego depletion, cognitive load, and time pressure may increase when self-control is low (Hofmann et al., 2009). Within this framework, negative emotions may indirectly undermine self-control. Self-control is considered a limited resource that enables the effortful regulation of dominant responses. This resource is depleted under conditions of ego depletion, which in turn impairs the performance of self-control (Hagger et al., 2010). Therefore, when adolescents perceive difficulties such as academic stress or experience negative emotions due to internal or external influences, their capacity for self-control may be diminished. Therefore, in order to prevent pathological gaming among adolescents, it is important to acknowledge the limited nature of their self-control resources and to implement strategies that mitigate the effects of ego depletion, thereby supporting their capacity to maintain self-regulation.

This study highlights that aggression should be considered one of the key factors to address in efforts to reduce pathological gaming among adolescents. Since aggression is related to negative emotions, self-control, and pathological gaming, it is an important factor. As shown in the results of this study, managing negative emotions during adolescence should be the starting point for reducing aggression. However, when presenting the goals to be achieved by managing negative emotions, while the reduction of aggression is important, the more focus should primarily be on self-control. Although negative emotions were not found to directly affect self-control, as discussed earlier, negative emotions influence academic stress and aggression, both of which are reasonably associated with self-control.

Thus, this study can provide the following suggestions to caregivers of adolescents or policymakers involved in pathological gaming-related issues. According to Achenbach and Edelbrock (1983), problematic behaviors related to emotional development in adolescents are largely categorized into internalizing and externalizing issues. Given that adolescents' externalized aggression is associated with anxiety and depression, it can be predicted that even if negative emotions are a natural part of adolescent development, long-term exposure without intervention may increase aggression and potentially lead to pathological gaming. Therefore, policies that focus on managing adolescents' negative emotions and aggression may be more effective in preventing such issues.

More importantly, the finding that self-control may partially mediate the relationship between aggression and pathological gaming suggests that supporting adolescents in maintaining self-control, even in the presence of increasing aggression, may help prevent the onset of pathological gaming. Additionally, this study supports prior research (Cao et al., 2007; Özdemir et al., 2014) that low levels of self-control are associated with impulsive behaviors and a tendency to seek immediate gratification, which can lead to behaviors such as pathological gaming. Furthermore, one of our key findings—that negative psychological states (anxiety, loneliness, depression, academic stress) can influence self-control—suggests that, in addressing pathological gaming in adolescents, it is important to consider both internal psychological states and environmental factors, which broadens the scope of existing discussions.

However, this study has several limitations. First, the study participants were all adolescents from Korea. Due to the competitive nature of education in Korea, particularly regarding university entrance, the findings may differ if the same research model is applied in regions or countries with different educational or cultural contexts. Furthermore, as this study was conducted with Korean adolescents, there are limitations in generalizing the influence of cultural factors on pathological gaming. Future research should examine the moderating effects of cultural differences on relevant variables by comparing individualistic and collectivistic cultural contexts. And, all measurements were based on self-reported data from the participants. While validated items from prior studies were used to measure the factors involved, there may be slight discrepancies between the participants' actual states and their self-reported data. And this data include adolescents across different school levels, future studies should account for potential developmental differences by controlling for school level or analyzing them within the model. Therefore, future research could measure these factors in different cultural or regional settings, or adopt other measurement systems, which could help expand the scope of research on adolescents' pathological gaming.

In addition, although PLS-SEM was conducted, the analysis primarily focused on hypothesis testing, and model fit indices such as the SRMR were not reported. Therefore, future research should include such indices to enable a more rigorous and comprehensive evaluation of the model. In this study, participant attrition occurred across certain time points due to the longitudinal design, and thus the possibility of dropout bias cannot be entirely ruled out. However, follow-up analyses of key demographic variables and average daily game usage time revealed no significant differences across time points (T1–T3). Although this study utilized a carefully constructed longitudinal dataset with minimal attrition bias as supported by follow-up analyses, sensitivity analyses were not performed separately. Therefore, the possibility that attrition may have affected the results cannot be entirely ruled out. Future research should consider incorporating sensitivity analyses or alternative methods such as multiple imputation to enhance the robustness of findings. Nevertheless, potential influences of unmeasured differences between completers and non-completers should be acknowledged, and future research would benefit from more systematic sample management and examination of dropout-related factors.

## 6 Conclusion

This study was conducted to empirically examine whether self-control mediates the relationships between adolescents' negative emotions, academic stress, aggression, and pathological gaming. The findings indicate that adolescents' negative emotions significantly influence academic stress and aggression, and that self-control fully or partially mediates the relationships between academic stress, aggression, and pathological gaming. Considering the increasing attention on adolescents' digital behaviors over time, focusing on managing negative emotions and enhancing self-control in a sustainable manner may serve as a practical approach to prevention. Our findings contribute to promoting healthy gaming behaviors among adolescents and developing relevant policies.

## Data Availability

Publicly available datasets were analyzed in this study. This data can be found here: https://www.kocca.kr/gameguide/subPage.do?menuNo=203709.

## Footnotes

^1^No significant differences were observed across T1, T2, and T3 in gender (χ^2^(2) = 1.252, *p* = 0.535), school level [χ^2^_(4)_ = 3.932, *p* = 0.415], or average daily gaming time [*F*_(2, 3644)_ = 0.010, *p* = 0.990].
